# A safe and effective micro-choice based rehabilitation for patients with long COVID: results from a quasi-experimental study

**DOI:** 10.1038/s41598-023-35991-y

**Published:** 2023-06-09

**Authors:** Bente Frisk, Marte Jürgensen, Birgitte Espehaug, Kiri Lovise Njøten, Eirik Søfteland, Bernt Bøgvald Aarli, Gerd Kvale

**Affiliations:** 1 Department of Health and Functioning, Western Norway University for Applied Sciences, Bergen, Norway; 2 Helse i Hardanger, Øystese, Norway; 3 Divison of Psychiatry, Haukeland University Hospital, PO Box 1400, 5021 Bergen, Norway; 4 Department of Medicine, Haukeland University Hospital, Bergen, Norway; 5 Department of Clinical Science, University of Bergen, Bergen, Norway; 6 Department of Thoracic Medicine, Haukeland University Hospital, Bergen, Norway; 7 Department of Clinical Psychology, University of Bergen, Bergen, Norway

**Keywords:** Infectious diseases, Patient education, Fatigue

## Abstract

At least 65 million people suffer from long COVID. Treatment guidelines are unclear, especially pertaining to recommendations of increased activity. This longitudinal study evaluated safety, changes in functional level and sick leave following a concentrated rehabilitation program for patients with long COVID. Seventy-eight patients (19–67 years) participated in a 3-day micro-choice based rehabilitation program with 7-day and 3-month follow-up. Fatigue, functional levels, sick leave, dyspnea and exercise capacity were assessed. No adverse events were reported and 97.4% completed the rehabilitation. Fatigue measured with Chalder Fatigue Questionnaire decreased at 7-days [mean difference (MD = − 4.5, 95% CI − 5.5 to − 3.4) and 3-month (MD = − 5.5, 95% CI − 6.7 to − 4.3). Sick leave rates and dyspnea were reduced (p < 0.001) and exercise capacity and functional level increased (p < 0.001) at 3-month follow-up regardless of severity of fatigue at baseline. Micro-choice based concentrated rehabilitation for patients with long COVID was safe, highly acceptable and showed rapid improvements in fatigue and functional levels, sustaining over time. Even though this is a quasi-experimental study, the findings are of importance addressing the tremendous challenges of disability due to long COVID. Our results are also highly relevant for patients, as they provide the base for an optimistic outlook and evidence supported reason for hope.

## Introduction

Following the SARS-CoV-2 pandemic, we are now hit hard by a new wave, namely long COVID (post COVID-19 condition)^1,2^. Months or years after the infection up to 50% of patients face persistent symptoms, with fatigue as one of the most commonly reported, impacting their level of functioning, quality of life and ability to work^1,3–5^. Young adults as well as non-hospitalized patients are also affected^1,5–7^, resulting in huge societal costs^8^.

Worldwide, health care providers and researchers are scrambling for ways to help these patients in getting back their ordinary lives. Several studies on rehabilitation have been published^9–17^, however, no consensus or “gold standard” for treatment recommendations exists^18^. One cross-sectional study^19^ which was given substantial weight in a recently published review article^18^, even indicated that only 1% of patients with long COVID benefited from increased physical activity, and this study also reported that for 75% of the patients, physical activity worsened the symptoms^19^. Given this pessimistic, although, insufficient scientifically founded perspective, there is an urgent need to share research that might give nuances to this grim picture.

The group behind this study has developed a comprehensive concentrated interdisciplinary group rehabilitation program for long COVID, which is described in detail in the protocol paper^20^. One of its main features is a shift in focus from targeting symptoms to targeting and monitoring seemingly insignificant micro-choices that facilitate increased flexibility and levels of functioning. The approach has shown effectiveness across a number of different chronic health challenges^21–24^.

The aims of the study were to assess safety, acceptability, potential changes in fatigue, sick leave, functional level, dyspnea, and exercise capacity from pre-treatment to 3 months follow-up and to explore predictors for change in fatigue.

## Methods

### Study design and participants

This quasi-experimental study with 3-month follow-up is part of the “Project Development of Smarter Health Solutions” (PUSH project), Haukeland University Hospital (HUH: Bergen, Norway) and Helse i Hardanger (HiH: Øystese, Norway)^20^.

Patients were referred to the Department of Thoracic Medicine, HUH, by their general practitioner, or other physicians. Eligible patients were adults between 18 and 67 years, with long COVID, defined as confirmed SARS-CoV-2 infection and persisting symptoms at least 3 months from the onset of the infection leading to impaired everyday functioning which could not be explained by alternative diagnoses^2^. Patients who had improved from long COVID when waiting for treatment were excluded. Diseases where physical activity was not recommended was also reason for exclusion. Participants had to be fluent in oral and written Norwegian and have sufficient digital competence to handle online questionnaires.

### Intervention

**Figure 1:**
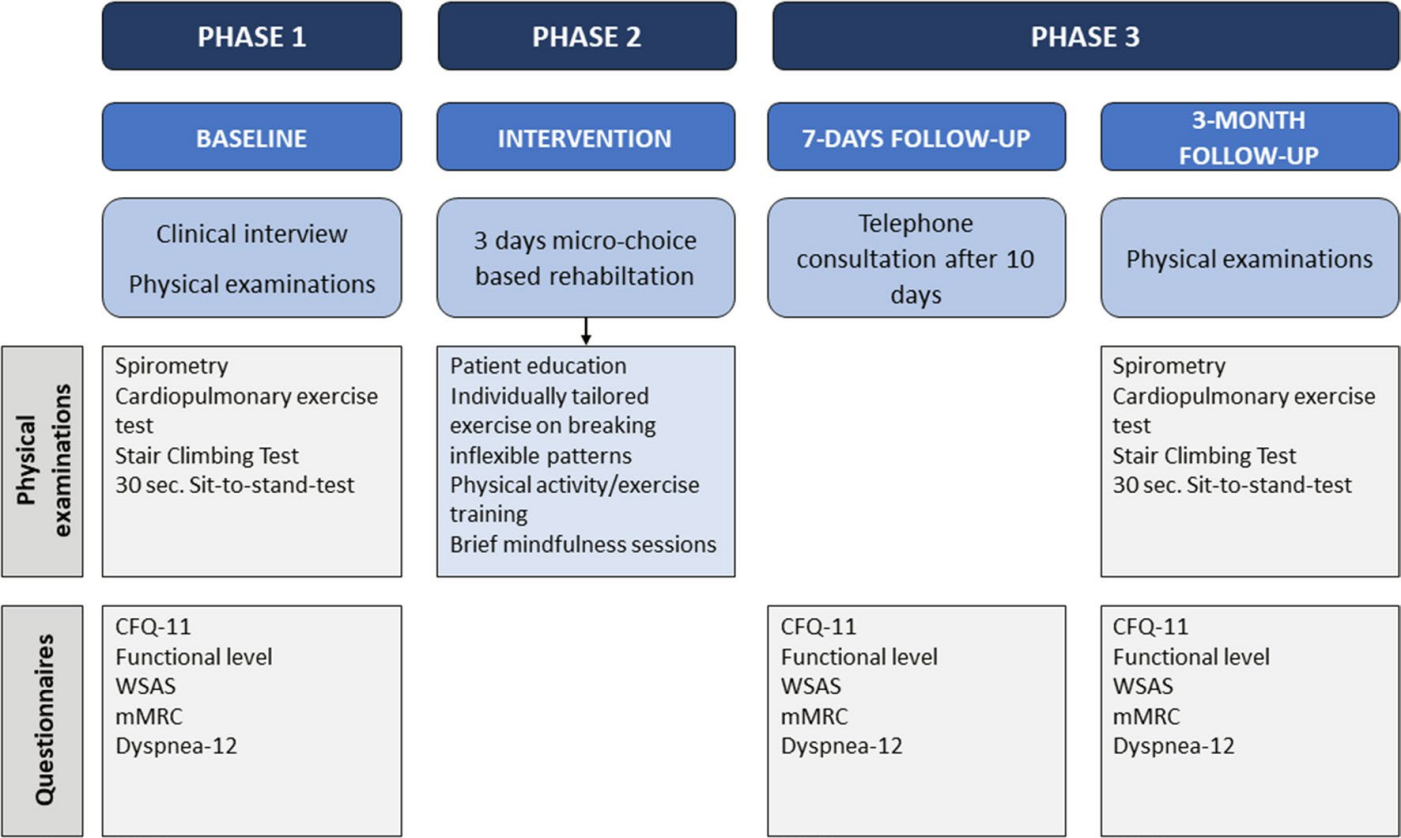
Timeline of the study. *CFQ* Chalder Fatigue Questionnaire, *WSAS* Work and Social Adjustment Scale, *mMRC* modified Medical Research Council dyspnea scale.

The clinical intervention consisted of three equally important phases (Fig. 1) and is outlined in a protocol paper for concentrated interdisciplinary group rehabilitation for patients with chronic illnesses^20^. Additionally, the specific questionnaires for fatigue and dyspnea, in addition to lung function and exercise measurements used in this study is not described in the generic protocol paper^20^.

#### Phase 1: Pre-treatment preparation

Standardized information about the intervention was given and physical examinations were done to exclude other medical conditions and to examine functional status (Fig. 1).

#### Phase 2: The concentrated micro-choice based rehabilitation

The core element in this out-patient rehabilitation, delivered in groups of 6–10 patients during three consecutive days (8.30–16.00), was a shift in focus, from targeting and monitoring symptoms, to focus on micro-choices in order to facilitate increased levels of physical activity and functioning (Fig. 1).

#### Phase 3: Integrating the changes into everyday living

The first 3 weeks after the concentrated rehabilitation, patients answered two questions digitally once a day (0–100) regarding strategies for handling symptoms: (1) *To* *what* *extent* *did* *you* *allow* *the* *symptoms* *to* *decide* *today*, and (2) to *what* *extent* *did* *you* *make* *use* *of* *the* *principle* *of* *doing* *something* *else*. An individual telephone consultation was conducted 10 days after the intervention (Fig. 1).

### Measurements

An overview of the measurement tools and the respective assessment times are presented in Fig. 1.

*Chalder* *Fatigue* *Scale* *(CFQ-11)* was used to assess mental and physical fatigue^25^.

CFQ-11 was calculated with total score (0–33) and bimodally (0–11). The bimodal score provides a method for distinction between “cases” and “non-cases”^26^. A bimodal score of ≥ 4 was defined as a case of fatigue^25,26^. Severe fatigue was calculated as a bimodal score ≥ 4 and total score ≥ 23^6^. Minimal clinically important difference (MCID) for total score is between 1.4 and 4^27^.

*Sick* *leave* *and* *%* *degree* *of* *sick* *leave* were registered digitally by the participants.

*The* *Work* *and* *Social* *Adjustment* *Scale* *(WSAS)*^28^ was used to assess functional level. Total score ranges from 0 to 40, where higher scores indicate functional impairment. The MCID is a reduction of 3.6 points^29^. In addition a *visual* *analogue* *scale* *(VAS)* (0–100) was used^30^. Patients were asked “If 100 represents your functional level before COVID-19, what is your functional level today?”.

*Modified* *Medical* *Research* *Council* *dyspnea* *scale* *(mMRC)*^31^ and *Dyspnea-12* were used to measure dyspnea^32^. A cut-off ≥ 1 for mMRC was used. MCID for Dyspnea-12 ranges between -3 and -6 points^33^.

*Ongoing* *psychiatric* *symptoms* and *previous* *psychiatric* *illness* were evaluated by a psychiatrist based on the clinical interview at baseline and information from medical records.

*Spirometry* was conducted on a Vyntus Body/APS Plethysmograph (Vyaire medical GmbH, Hochenberg, Germany) according to the American Thoracic Society/European Respiratory Society guidelines^34^.

*Cardiopulmonary* *exercise* *test* *(CPET)* was performed by uphill walking on a treadmill until exhaustion (Woodway, Würtzburg, Germany). Gas exchange and ventilatory variables were measured by breath-by-breath sampling averaged over 30 s intervals (Hans Rudolph two way breathing mask: V2 mask, Shawnee, USA). Heart rate was measured through a 12-lead electrocardiogram ECG (Custo Cardio 300, custo med, Ottobrun, Germany), and oxygen saturation (SpO_2_) with an ear probe using a stationary pulse oximeter (Xpod, Nonin, Minnesota, USA). Blood pressure was measured with Tango M2 (SunTech Medical, Morrisville, USA). The walking speed was set individually with an inclination at 0% at start with increased inclination every 60 s by 2% up to 20%. Thereafter the speed was increased by 0.5 km h^−1^ until exhaustion. Reasons for termination were pronounced pain or dizziness, ischaemic ECG changes or decreased systolic blood pressure below the resting pressure^35^. Dyspnea and leg fatigue were measured with Borg CR10 scale^36^. All assessments were measured at rest, throughout the test (Borg CR10 every second minute), at peak exercise and two minutes after termination of the test. Norwegian reference values for CPET variables were used^37^. Breathing reserve was calculated as maximal ventilatory limitation (MVV −peak ventilation $\dot{V}$_Epeak_/MVV × 100) using an estimate for MVV as forced expiratory volume in 1 s FEV_1_ × 40^38^. Ventilatory limitation was defined when breathing reserve < 15%^38^. Reduced exercise capacity was defined as peak oxygen uptake $\dot{V}$O_2peak_ < 85% predicted^38^. CPET was considered maximal if respiratory exchange ratio RER ≥ 1.1.

*Stair* *Climbing* *Test* *(SCT)* was used to assess submaximal exercise capacity and *30-s* *sit-to-stand-test* *(30STST)* to assess lower extremity strength^39^. MCID is not reported for SCT and 30STST for patients with long COVID.

### Statistical analyses

Data were analysed with IBM SPSS Statistics version 28 (SPSS Inc., Chicago, USA) and Stata version 17 (StataCorp). Descriptive statistics were used to characterize the study population (mean, standard deviation (SD), median, and percent). Logistic mixed effect models were used to estimate change from pre-treatment to 3-month follow-up in sick leave, and linear mixed effect models to estimate change in CFQ-11, Dyspnea-12, functional level, WSAS, 30STST, SCT and $\dot{V}$O_2peak_ kg^−1^. The regression models were fitted with random intercepts and random slopes for time. Assumptions were checked with diagnostic plots. To explore predictors for change in CFQ-11, interaction terms between time and gender, age, time since infection, psychiatric illness, functional level and $\dot{V}$O_2peak_ kg^−1^, respectively, were included in the models. A global test was performed to assess statistically significant interaction terms at 7-days and 3-month follow-up. Paired samples t-tests were used to analyse the change in CPET variables, lung function and Wilcoxon signed-rank test to analyse change in mMRC. Normality of change-data was checked by histograms, QQ-plots and Shapiro–Wilk’s test. Estimated changes from baseline to follow-up are presented with 95% confidence intervals (CI) and p values. Statistical significance was set at α = 0.05.

The data were collected electronically and by physical examinations and were stored on an encrypted server at Western Norway Regional Health Authority IKT.

### Ethical approval

The PUSH project and the specific study protocol^20^ for patients with long COVID included in the current study were approved by the Western Norway Regional Committees for Medical and Health Research Ethics (REK 2020/101648), and is registered in Clinical Trials (NCT05234281, approval date: 10/02/2022). Informed consent was obtained from all participants included in the study. All methods were performed in accordance with the relevant guidelines and regulations.

## Results

### Safety of the intervention and baseline characteristics

**Figure 2:**
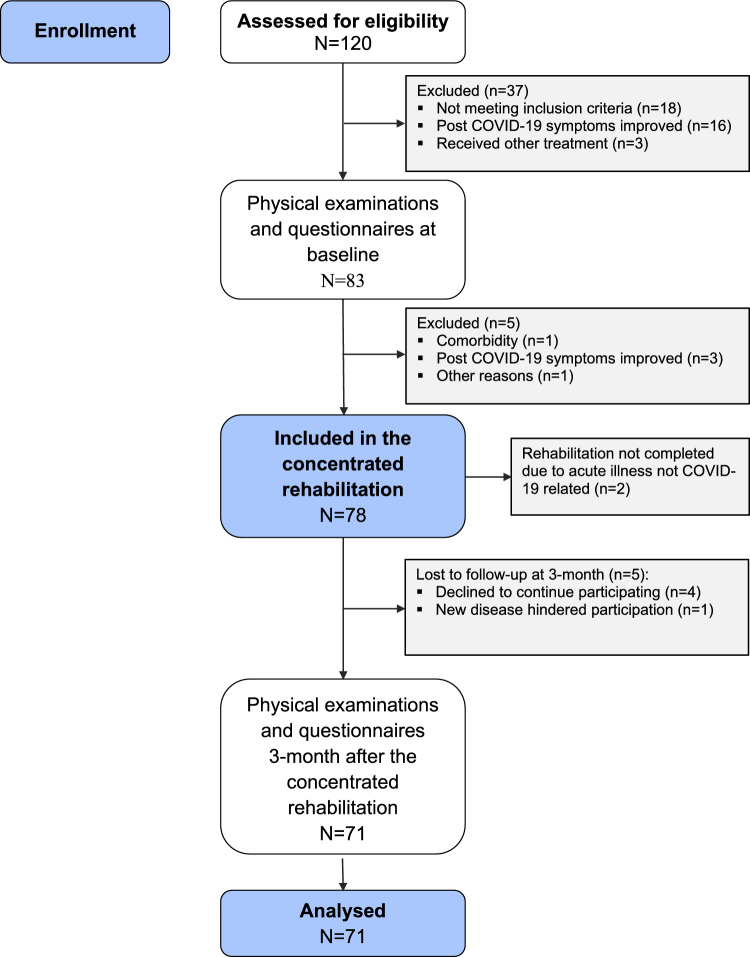
Flowchart of the study.

A total of 120 patients were assessed for eligibility to the intervention, of which 78 met the inclusion criteria (Fig. 2) and all accepted participation. The completion rate was 97.4% and no adverse events were reported (Fig. 2). Two patients (2.6%) reported slightly reduced levels of functioning at 7-days after the intervention, but at 3-month follow-up the functional level was improved. One patient (1.2%) reported reduced levels of functioning at 3-month. Table 1 summarises baseline characteristics. Mean duration of symptoms were 10.2 months, with fatigue (99%) and dyspnea (63%) as most frequently reported. Obesity (BMI > 30 kg m^−2^) was present in 16 (21%) participants, and 14 (18%) had been hospitalised during the acute infection, 3 (4%) needing intensive care treatment. For some of the measurements there were some missing data due to technical problems, pain or other symptoms that hampered completion of the physical tests or questionnaires in the mobile application. We report the exact number analysed for each measurement.

**Table 1:** Baseline characteristics.

Variables	Total N = 78
Sex, female n (%)	64 (82)
Age at baseline (years)	40.3 ± 12.0
Height (m)	1.71 ± 0.08
Weight (kg)	78.2 ± 16.0
BMI (mean, kg m^−2^)	26.7 ± 5.0
Marital status, n (%)	
Single	21 (27)
Married/cohabitant	52 (67)
Girl-/boyfriend	5 (6)
Education, n (%)	
Lower secondary	1 (1)
Upper secondary	19 (24)
Higher	58 (75)
Working status, n (%)	
Employee	65 (83)
Student	10 (13)
Disability benefit	2 (3)
Pensioner	1 (1)
Employment rate before onset of COVID-19 median (min–max)	100 (10–100)
Sick leave at inclusion (yes) n (%)	39 (63)
Sick leave rate at inclusion (%)	51.0 ± 42.1
COVID-19 specific characteristics, n (%)	
Mild/Moderate	64 (82)
Critical	11 (14)
Severe	3 (4)
Time to rehabilitation after confirmed COVID-19 (months)	10.2 ± 4.8
Signs and symptoms of long COVID, n (%)	
Dyspnea	49 (63)
Fatigue	77 (99)
Feeling depressed	26 (33)
Other	40 (51)
Psychiatric illness, n (%)	
None	42 (54)
Previous psychiatric illness	19 (24)
Ongoing anxiety and/or depression	17 (22)

Data are presented as mean ± standard deviation (SD) and percent otherwise stated.

### Changes in fatigue and predictors for change

**Figure 3:**
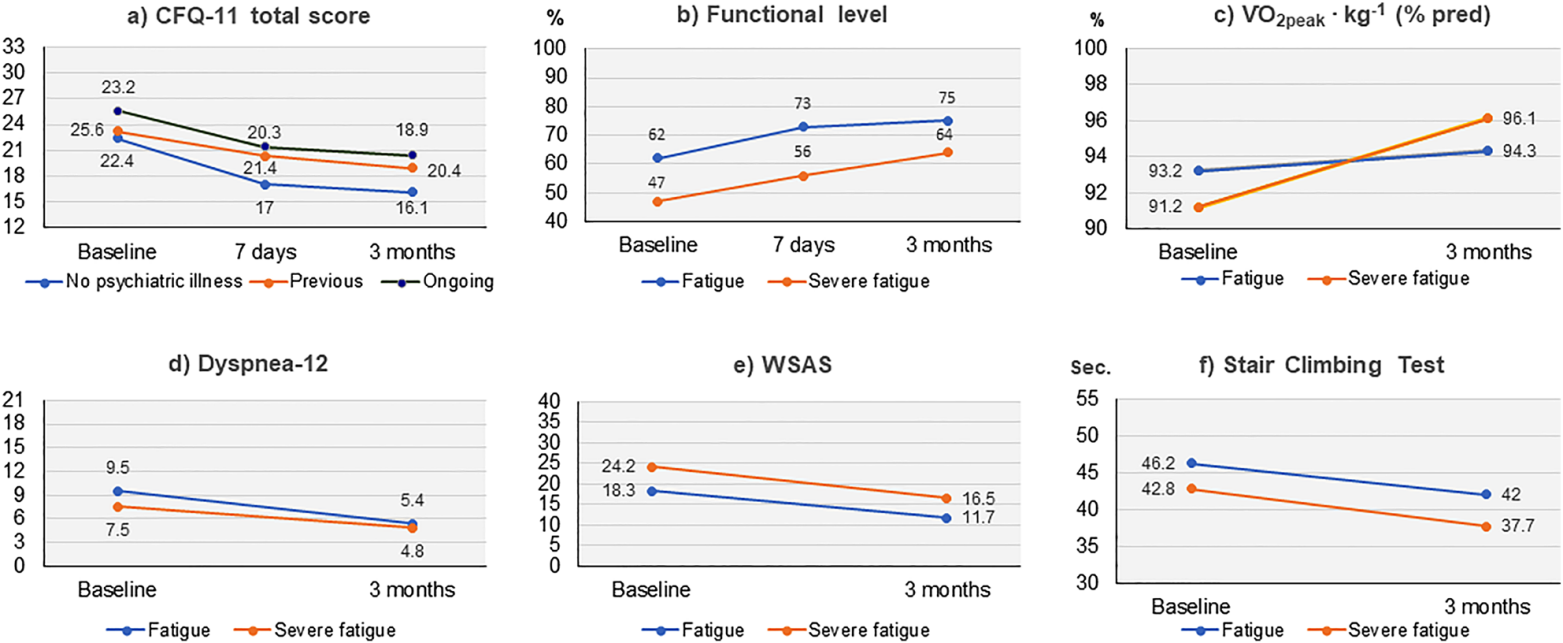
Changes in fatigue, functional status, exercise capacity and dyspnea from baseline to 3-month follow up. (**a**) The unadjusted mean values are presented for patients with no, previous or ongoing psychiatric illness, respectively, and in (**b**–**f**) the unadjusted mean values are presented for patients with fatigue (a bimodal CFQ-11 score ≥ 4), and severe (bimodal CFQ-11 score ≥ 4 and total score ≥ 23). The four patients with no fatigue at baseline are included in the fatigue group. *CFQ* Chalder Fatigue Questionnaire, $\dot{V}$*O*_*2peak*_ peak oxygen uptake, *WSAS* Work and Social Adjustment Scale.

**Figure 4:**
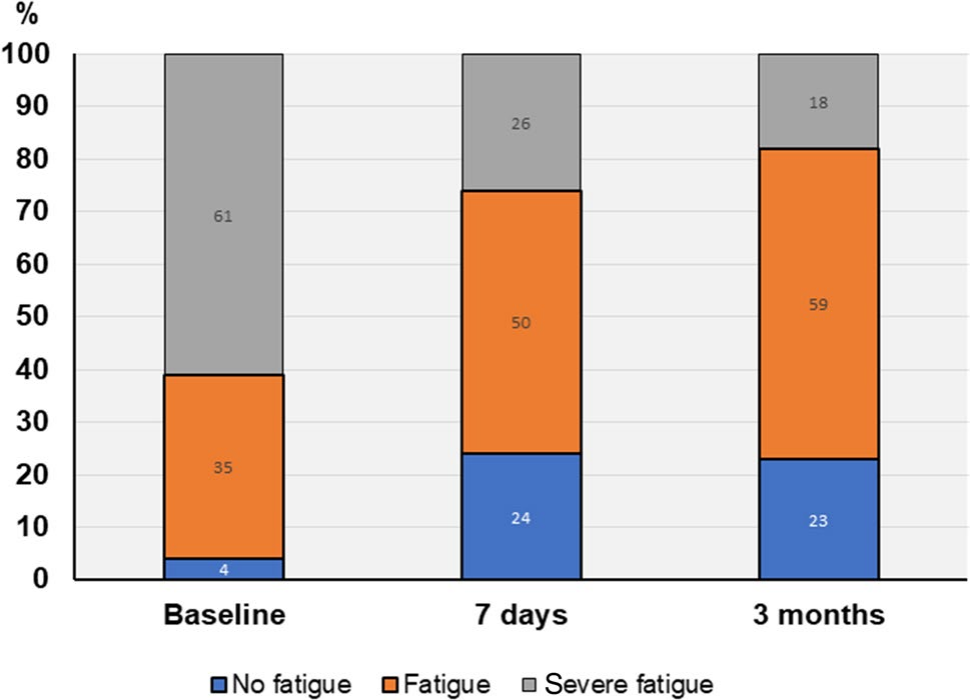
Changes in fatigue severity from baseline to 3-month follow-up.

Compared with baseline measures, mean CFQ-11 was reduced with 4.5 points (p < 0.001) at 7-days and 5.5 points at 3-month (p < 0.001) (Table 2). The reduction was larger than MCID. One patient reported an increased CFQ-11 larger than MCID at 7-days, however at 3-month symptoms of fatigue was clinically significant improved. Global tests of interactions showed that overall, the observed reduction in CFQ-11 was not modified by any of the investigated predictors (p ≥ 0.2). Accordingly, the decline in mean CFQ-11 was observed in participants irrespective of ongoing or previous psychiatric illness, or never experienced psychiatric illness (Fig. 3). Mean bimodal score was 8.8 (SD 2.2) at baseline, with reductions to 6.8 (SD 3.8) and 6.1 (SD 3.5) at 7-days and 3-month, respectively. At baseline, 61% of the patients had severe fatigue (Fig. 4). The proportion with severe fatigue was reduced to 26% at 7-days and 18% at 3-month follow-up, whereas 24% at 7-days and 23% at 3-month, did not meet the criteria for fatigue (Fig. 4).

**Table 2:** Changes in fatigue, sick leave, functional level, exercise capacity and dyspnea from baseline to 3-month follow-up.

Outcome	Time	n	M (SD)^a^	Estimated change^c^			
				OR^b^	MD^c^	95% CI	p value
CFQ-11	Baseline	77	23.2 (4.5)				
	7-day	74	18.7 (6.6)		− 4.5	− 5.6 to − 3.4	< 0.001
	3-month	71	17.7 (5.7)		− 5.5	− 6.7 to − 4.3	< 0.001
Sick leave	Baseline	39	63^4^	1			
	3-month	23	43^4^	0.2		0.1 to 0.7	0.02
Functional level	Baseline^c^	75	52.9 (15.8)^4^				
	7-day	73	62.7 (16.3)^4^		10.0	6.8 to 13.2	< 0.001
	3-month	71	68.7 (18.0)^4^		15.8	11.9 to 19.6	< 0.001
WSAS	Baseline^c^	75	21.9 (8.1)				
	3-month	71	14.6 (9.6)		− 6.9	− 8.9 to − 4.9	< 0.001
30STST	Baseline^c^	75	19.0 (6.5)				
	3-month	68	22.6 (6.9)		3.3	2.3 to 4.2	< 0.001
SCT	Baseline^c^	77	44.1 (12.6)				
	3-month	66	39.6 (9.1)		− 3.8	− 4.7 to − 2.8	< 0.001
$\dot{V}$O_2peak_ kg^−1^	Baseline^c^	77	30.8 (6.2)		30.6		
	3-month	67	31.5 (6.4)		0.9	0.3 to 1.5	0.002
Dyspnea-12	Baseline^c^	68	7.8 (7.0)				
	3-month	71	4.6 (5.5)		− 3.3	− 4.8 to − 1.8	< 0.001

*M* mean, *SD* standard deviation, *CI* confidence Interval, *CFQ* Chalder Fatigue Questionnaire, *WSAS* Work and Social Adjustment Scale, *30STST* 30 s sit to stand test, *SCT* Stair Climbing Test, $\dot{V}$*O*_*2peak*_ peak oxygen uptake.

^a^Unadjusted mean values.

^b^Odds ratio (OR): estimated with logistic mixed effect regression with time in the model.

^c^Mean difference (MD) estimated with linear mixed effect regression with time in the model.

^4^Percent.

### Changes in sick leave and functional level

The proportion of employed participants on sick leave was reduced from 63% at baseline to 43% at 3-month (OR = 0.2, p = 0.02) (Table 2). Further, the mean degree of sick leave was reduced from 51 to 30%. The sick leave rate was reduced in those with fatigue from 42 to 22% (p = 0.019) and severe fatigue from 59 to 36% (p = 0.002) (not shown in table).

Self- reported functional level increased from 53% at baseline to 63% and 69% at 7-days and 3-month follow-up, respectively (p < 0.001) (Table 2). At baseline the mean WSAS score was 21.9 with a reduction of 6.9 at 3-month, which was larger than MCID (Table 2). The findings for functional level and WSAS for those with fatigue and severe fatigue were similar (Fig. 3).

### Changes in dyspnea, lung function and exercise capacity

At baseline 69.7% of the participants scored ≥ 1 on mMRC while at 3-month follow-up this was reduced to 57.7% (p = 0.03) (Table 3). Dyspnea-12 total score was reduced significantly with 3.3 points which is within the range of MCID between 3 and 6 points at 3-month (Table 2). For the first patient group there were technical problems in the mobile application which resulted in missing data storage for 10 patients at baseline.

**Table 3:** Changes in CPET variables, lung function and dyspnea from baseline to 3-month follow-up.

	Baseline		3-month		95% CI*	p value
	N	Mean (SD)	N	Mean (SD)		
CPET						
Exercise time (min, s)	77	9.0 (1.6)	68	9.3 (1.7)	− 0.65 to − 0.03	0.032
$\dot{V}$O_2peak_ (mL min^−1^)	77	2366 (514)	69	2426 (553)	− 125.1 to − 28.9	0.002
$\dot{V}$O_2peak_ (% predicted)	77	92 (17)	68	95 (18)	− 4.8 to − 1.3	< 0.001
$\dot{V}$O_2peak_ kg^−1^ (mL kg^−1^ min^−1^)	77	31 (6)	67	32 (6)	− 1.6 to − 0.4	0.002
$\dot{V}$O_2peak_ kg^−a^ (% pred)	76	84 (15)	65	88 (17)	− 4.2 to 0.1	0.066
Perceived dyspnea Borg CR10 at max load	75	8.4 (1.7)	68	8.6 (1.9)	− 0.76 to 0.07	0.101
Perceived leg discomfort Borg CR10 at max load	75	7.6 (2.0)	68	8.2 (2.3)	− 1.13 to − 0.02	0.043
Gas exchange						
SpO_2_ max load (%)	76	96.1 (3.6)	66	95.7 (3.8)	− 0.4 to 1.9	0.211
RER at max load	77	1.15 (0.09)	69	1.19 (0.09)	− 0.01 to − 0.01	0.004
Lung function						
FEV_1_% pred	78	96.6 (11.4)	69	97.5 (12.0)	0.6 to − 1.1	0.282
FVC % pred	78	100.3 (11.2)	69	100.6 (11.9)	1.4 to − 0.1	0.920
FEV_1_/FVC % pred	78	96.1 (7.6)	69	96.5 (7.0)	− 1.5 to 0.8	0.570
DLCO % pred	75	89.9 (24.3)	70	87.4 (12.3	− 2.6 to 8.7	0.285
Dyspnea						
mMRC < 1	23	30.3	30	42.3		
mMRC ≥ 1 (%)	53	69.7	41	57.7		0.003^a^

*CPET* cardiopulmonary exercise test, $\dot{V}$*O*_*2peak*_ oxygen uptake, *SpO*_*2*_ oxygen saturation, *RER* respiratory exchange ratio, *FEV*_*1*_ forced expiratory volume in 1 s, *FVC* forced vital capacity, *DLCO* diffusing capacity for carbon monoxide.

*95% CI confidence interval for changes from baseline to 3-month follow-up.

^a^Analysed with Wilcoxon Signed Rank test. All other analyses were done with paired sample t-tests.

Lung function was within normal values at baseline and 3-month (Table 3).

At baseline the participants had a mean $\dot{V}$O_2peak_ kg^−1^ of 92 (17) % of predicted value (Table 3), 41% had $\dot{V}$O_2peak_ < 80% predicted. At 3-month this was reduced to 32%. $\dot{V}$O_2peak_ kg^−1^ increased significantly (p = 0.002), and the group with very severe fatigue at baseline had a significantly larger improvement compared to the others (Fig. 3).

SCT and 30STST also showed significant improvement at 3-month follow-up (Table 2).

## Discussion

A highly pessimistic picture has been painted for patients suffering from long COVID. The current paper present results that are in clear contrast to this: Following a 3-day, micro-choice based group intervention, the patients’ level of functioning increased significantly and there was a rapid, significant, and clinically important reduction in fatigue at 3-month follow-up, in addition to significantly reduced dyspnea and improved exercise capacity. Mean pre-treatment symptom duration was 10.2 months. There were no indications of post-exertional malaise or other adverse events.

These results are important, both for health care workers and for patients, given that the current treatment guidelines are unclear, especially with regards to recommendations of increased activity. Strikingly, one cross-sectional study indicated that less than 1% of long COVID victims benefited from physical activity, with detrimental effects seen in 75%^19^. In clear contrast, the results of our study showed rapid, consistent and highly relevant improvements in fatigue, physical functioning and work participation, with no harmful effects. The approach may thus represent one answer to the call for evidence supported treatments for long COVID^18^. At 3-month follow-up the improvements in our study were sustained, in addition to significantly reduced dyspnea and improved exercise capacity.

A rapid return to work, as shown in our study, can reduce societal costs substantially and increase the quality of life for the individuals. The results are in line with findings from concentrated treatment for other conditions^21–24^, and this further development of the concentrated treatment format may thus have implications for the way we provide rehabilitation for patients with long COVID.

Most participants in our study had not been hospitalized during the acute phase, making these results relevant for the majority of patients suffering from long COVID^6,7^. Furthermore, the study population is comparable to a cohort of home-isolated COVID-19 patients recruited from the same geographical area in the same period^6,40^. In this cohort, fatigue was significantly higher compared to healthy controls and was not reduced 12 months after the acute infection. In comparison, our participants reported a much higher fatigue at baseline yielding a higher proportion of fatigue (96% vs 30%) and severe fatigue (61% vs 7%)^6^, indicating that our patients were severely affected by long COVID and that spontaneous recovery before participation was unlikely. In addition, the long duration of symptoms before the intervention makes it less likely that the rapid improvement was due to natural recovery and rather a result of the intervention. Our results are relevant for patients struggling with long COVID, as they provide the base for an optimistic outlook and evidence-based reason for hope.

The greatest proportion of fatigue reduction was seen rapidly, already 7 days after the intervention. The decrease was clinically significant and maintained after 3 months^27^. The large reduction in severe fatigue after 1 week, suggests that the intervention is particularly useful for the most severely affected. Reduction in fatigue has also been reported after other rehabilitation interventions^10,15–17^. Jimeno-Almazan et al.^15^ showed a large reduction in fatigue following an 8-week supervised exercise intervention compared to a control group.

The fact that we did not reveal any baseline predictors of change in fatigue indicates that the intervention may be beneficial to patients with long COVID, regardless of duration, age or gender. Furthermore, the findings imply that previous or ongoing psychiatric illnesses do not constitute barriers to improvement through concentrated rehabilitation and that these patient groups should therefore not be excluded. This might also be noted as interesting given the potential effects on mental health related to the lock-down^41–43^. In line with this, our group has previously found increased functional levels and decreased symptoms following concentrated rehabilitation for patients with longstanding anxiety and/or depression^24^.

Despite baseline $\dot{V}$O_2peak_ values within the normal range of predicted values, the participants significantly improved $\dot{V}$O_2peak_ at 3-month. We also observed an increase in SCT and 30STST. The increases were consistent in both maximal and submaximal exercise capacity as well as for lower limb strength. These findings are in line with other studies on rehabilitation for patients with long COVID^10–12,15,17^. While no significant differences in exercise capacity were found between those with fatigue and severe fatigue at baseline, patients with severe fatigue had a significantly larger increase in $\dot{V}$O_2peak_. This underlines that the intervention seems particularly useful in the most seriously affected. Adverse reactions to physical activity were closely monitored in our study. We found no indication of this occurring in our patient cohort, quite the contrary, as both self-reported activity levels as well as objective measures improved. Hence, our results support the notion of targeting the seemingly insignificant micro-choices in order to achieve a substantial increase in functional levels.

### Strengths and limitations

This study represents a novel way of providing rehabilitation for patients with long COVID, based on experiences from the concentrated treatment format^21–24^, and shows that a concentrated intervention may result in large changes in both symptoms and functional levels. Compliance with the intervention was high, and the results were highly significant and consistent across a number of subjective assessments of symptoms and function and objective tests examining exercise capacity. Due to the lack of a control group, it is not possible to rule out that the treatment effect can be non-specific and due to attention. However, due to long waiting time, we had the possibility to exclude those with spontaneous improvement, allowing only participants with persistent symptoms and no improvement to participate. The long duration of symptoms and reduced functional level before participation contrasts with the rapid improvement after the intervention. Hence, it seems unlikely that the changes observed were due to spontaneous recovery. Moreover, follow-up data at 3 months showed a sustained improvement over time. It might be noted that the complex intervention has elements from a number other approaches, for example the cognitive behaviour therapy for chronic fatigue syndrome^44^. However, the design in the current study does not allow for identifying the relative importance of the component(s).

The sample size was moderate, although larger than several comparable studies^10,15,17^. Further research should therefore be conducted to investigate transferability to larger group of patients with long COVID. However, further strengthening our conclusions, a similar format of concentrated rehabilitation has also been shown effective in reducing fatigue for patients with chronic fatigue syndrome^21^.

## Conclusion

This study of a micro-choice based 3-day concentrated group rehabilitation for long COVID yielded strong results. Rapid, sustained, and consistent improvements were observed for fatigue, dyspnea, sick leave, functional level, and exercise capacity. No safety issues were detected. The findings are in agreement with results of the concentrated treatment format for other chronic conditions and may be of importance for the large numbers of individuals worldwide experiencing persistent symptoms and disability due to long COVID.

## Data Availability

In accordance with the approvals granted for this study by the Regional Committee on Medical Research Ethics and the Norwegian Data Inspectorate, the data files will be stored securely and in accordance with the Norwegian Law of Privacy Protection. A subset of the data file with anonymized data will be made available to interested researchers upon reasonable request to Bente Frisk: bente.frisk@hvl.no, providing that Norwegian privacy legislation and the General Data Protection Regulation are respected, and that permission is granted from the Norwegian Data Inspectorate and the data protection officer at Haukeland University Hospital.
